# Heterogeneity in defining multiple trauma: a systematic review of randomized controlled trials

**DOI:** 10.1186/s13054-023-04637-w

**Published:** 2023-09-22

**Authors:** Thomas Jeanmougin, Elaine Cole, Baptiste Duceau, Mathieu Raux, Arthur James

**Affiliations:** 1 Sorbonne University, GRC 29, AP-HP, DMU DREAM, Department of Anesthesiology and critical care, Pitié-Salpêtrière Hospital, Paris, France; 2 Centre of Trauma Sciences, Blizard Institute, Queen Mary University of London, London, UK; 3 Sorbonne Université, INSERM, UMRS1158 Neurophysiologie Respiratoire Expérimentale et Clinique; AP-HP, Groupe Hospitalier Universitaire APHP-Sorbonne Université, site Pitié-Salpêtrière, Département d’Anesthésie Réanimation, F-75013 Paris, France

## Abstract

**Introduction:**

While numerous randomized controlled trials (RCTs) have been conducted in the field of trauma, a substantial portion of them are yielding negative results. One potential contributing factor to this trend could be the lack of agreement regarding the chosen definitions across different trials. The primary objective was to identify the terminology and definitions utilized for the characterization of multiple trauma patients within randomized controlled trials (RCTs).

**Methods:**

A systematic review of the literature was performed in MEDLINE, EMBASE and clinicaltrials.gov between January 1, 2002, and July 31, 2022. RCTs or RTCs protocols were eligible if they included multiple trauma patients. The terms employed to characterize patient populations were identified, and the corresponding definitions for these terms were extracted. The subsequent impact on the population recruited was then documented to expose clinical heterogeneity.

**Results:**

Fifty RCTs were included, and 12 different terms identified. Among these terms, the most frequently used were “*multiple trauma”* (*n* = 21, 42%), *"severe trauma"* (*n* = 8, 16%), *"major trauma"* (*n* = 4, 8%), and *trauma with hemorrhagic shock"* (*n* = 4, 8%). Only 62% of RCTs (*n* = 31) provided a definition for the terms used, resulting a total of 21 different definitions. These definitions primarily relied on the injury severity score (ISS) (*n* = 15, 30%), displaying an important underlying heterogeneity. The choice of the terms had an impact on the study population, affecting both the ISS and in-hospital mortality. Eleven protocols were included, featuring five different terms, with "severe trauma" being the most frequent, occurring six times (55%).

**Conclusion:**

This systematic review uncovers an important heterogeneity both in the terms and in the definitions employed to recruit trauma patients within RCTs. These findings underscore the imperative of promoting the use of a unique and consistent definition.

**Supplementary Information:**

The online version contains supplementary material available at 10.1186/s13054-023-04637-w.

## Introduction

Trauma is an important health issue worldwide, with an estimated 50 million individuals injured every year. It is a leading cause of death and disability especially in people under 40 years old [1, 2]. For these reasons, many randomized controlled trials have been conducted aiming to improve health outcomes after trauma. However, despite high expectancies, many of these trials have yielded negative results [3–6]. The impact of negative trials is an important issue for clinicians, patients and funders [7, 8]. Different reasons have been suggested to explain negative results in trials, such as lack of power, issues related with complex interventions or population heterogeneity [9–11]. Another potential reason is the lack of an accurate definition of the medical condition under investigation within a trial, especially in time-sensitive situations like trauma where early inclusions may be required despite uncertain or incomplete information.

In 2009, Butcher et al. suggested in a systematic review that at least 47 different definitions were coexisting in the published literature for multiple trauma patients [12]. Yet, among all trauma populations, those with the most severe injuries require a clear definition to ensure consistency across studies. In 2014, an international consensus proposed a unique definition and identified “polytrauma” patients as those with *significant injuries of three or more points in two or more different anatomic Abbreviated Injury Score (AIS) regions in conjunction with one or more additional variables such as a systolic blood pressure inferior or equal to 90 mmHg, a Glasgow score inferior or equal to 8, a base excess inferior or equal to -6 mmol/L, an international normalized ratio superior or equal to 1.4 or an age superior or equal to 70 year* [13]. Despite the involvement of international experts and a rigorous methodological process, this definition remains a challenge, particularly in studies that focus on prehospital care or early in-hospital care, where Injury Severity Score (ISS) cannot be determined until CT scan is performed and interpreted [14].

We hypothesized that varying definitions continue to be employed, which can result in heterogeneity across trauma randomized controlled trials (RCTs). The primary objective was to perform a systematic review to identify the terminology and definitions utilized for the characterization of multiple trauma patients within published and ongoing randomized controlled trials (RCTs).

### Methods

This systematic review follows the Preferred Reporting Items for Systematic Reviews and Meta-analyses (PRISMA) [15] (Additional file 1: Material 1).

#### Terms and definition

We defined as the *terms* all the synonyms used in RCTs to name these most severely injured patients (e.g., “multiple trauma”, “polytrauma” or “severe trauma”) and as the *definition* as the criteria reported to circumscribe each term (e.g., “Injury Severity Score (ISS) > 15”).

To ensure consistency in this systematic review, we adopted a single term and chose to use “multiple trauma” to align with the existing Mesh Terms thesaurus that have been utilized in PubMed since 1988 [16].

#### Data source and search strategy

A comprehensive search was conducted in MEDLINE and EMBASE using standardized vocabulary and free text to identify RCTs including multiple trauma patients published between January 1, 2002, and July 31, 2022. To ensure the broader recruitment among randomized controlled studies including multiple trauma patients, we used in the search equation a wide range of terms such as *multiple trauma, polytrauma*, *severe trauma* or *multiple injuries*. In addition, we also searched *ClinicalTrials.gov* to identify protocols of ongoing trials including multiple trauma patients. Details of the search strategy are provided in (Additional file 1: Material 2).

#### Inclusion and exclusion criteria

Eligibility criteria were RCTs or protocol for RCTs that included or aimed to include adult patients with multiple trauma. We excluded RCTs with less than 20 patients in the intervention arm and considered the arm with the smallest number of patients for RCTs with more than one intervention arm. We excluded RCTs recruiting only traumatic brain injury patients, post hoc analysis of previously published RCTs and publications in language other than English.

After identification and exclusion of duplicates, two reviewers (T.J & A.J) independently examined titles and abstracts to assessed eligibility of retrieved reports. All disagreements were resolved by discussions.

#### Data extraction

A standardized data collection form was used to extract the following information from full-text reports: study characteristics (name, first author, country of the first authors, year of publication, name of the journal, multicentric, inclusion criteria, verbatim description of both arms [intervention(s) and control] and main outcome), characteristics of the population included (number of patients, age, sex ratio, ISS), outcomes (mortality, main outcome significance as reported by the p-value) and finally the *terms* and *definitions* used to describe multiple trauma patients in these studies.

#### Risk of bias assessment

One reviewer (T.J) extracted the risk of bias from each full text included using the revised risk of bias assessment tool for randomized trial (RoB2) [17] and thus focused on five categories: the randomization process, the potential deviations from intended interventions, missing outcome data management, outcomes measurement process and the selection in reported results. The overall risk was thus determined from these five categories and reported for each published study.

#### Statistical analysis

The quantitative data were reported either as mean and standard deviation, or as minimum and maximum values. When the mean value was not available in full-text article, we use a method developed by Luo et al. to estimate the mean and standard deviation from median and quantiles [18]. Categorical data were described as counts and percentage. The thresholds used to qualify the included RCT as significant are those determined by the authors of the studies. We did not contact the trials authors for missing information. Statistical analyses and figures were performed with Python (v3.10.7).

## Results

### Search results

For published RCTs, after removing duplicates, 1699 studies were retrieved from the search. Of these, 1621 were excluded after title and abstract assessment. Following the initial screening process, the full texts of the 78 remaining studies were evaluated for eligibility. Of these, 10 studies were excluded due to insufficient sample size, eight studies did not involve multiple trauma patients, five studies involved post hoc analysis of other RCTs, four studies were not available in English language, and one study was identified as a duplicate. Consequently, 50 RCTs met inclusion criteria after rigorous full-text examination.

**Fig. 1:**
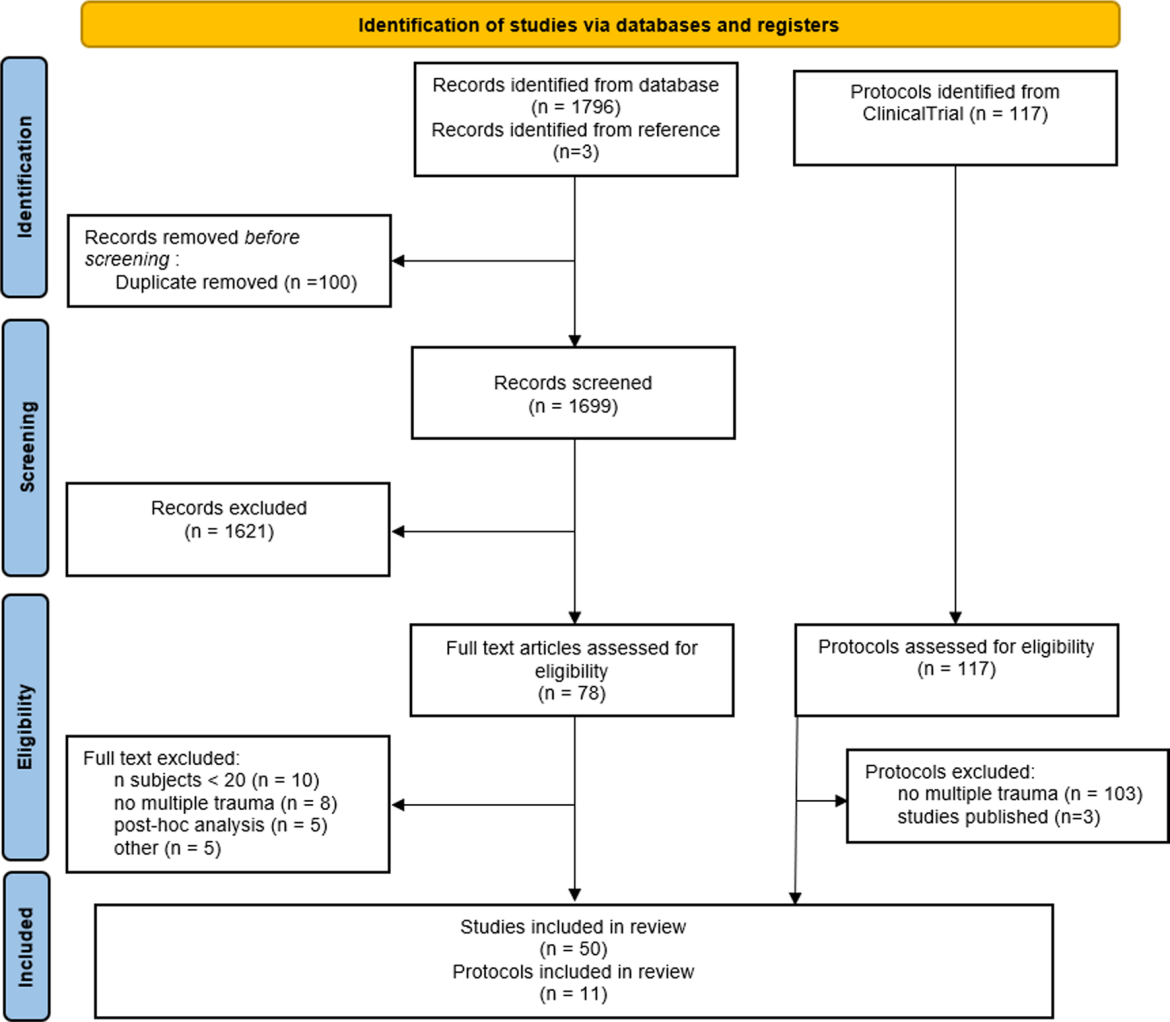
Systematic review flowchart

For protocols, 117 reports were obtained from clinicaltrials.gov for potential inclusion in the study protocols. After conducting a comprehensive screening process, 103 reports that did not target multiple trauma patients were excluded. Of the 14 remaining reports, three were found to be related to previously published RCTs, leaving a total of 11 which met inclusion criteria (Fig. 1).

### Characteristics of the RCTs

Table 1 provides a summary of the key characteristics of the included studies, with detailed information available in (Additional file 1: Material 3). Among these studies, 46% were multicentric (*n* = 23) and 56% (*n* = 28) involved a pharmacological intervention, while others were targeting a non-pharmacological treatment. In pharmacological RCTs, the control arm involved a placebo in 39% of the studies (*n* = 11/28).

**Table 1:** Characteristics of included studies (RCTs)

	All studies *n* = 50	Studies that provide a definition *n* = 31	Studies that do not provide a definition *n* = 19
Number of patients included, mean (min – max) Studies including more than 100 patients, *n *(%)	233 (41–1629) 27 (54)	296 (45–1629) 20 (65)	128 (41–573) 7 (37)
Age, years min – max	31–70	31–70	32–46
ISS, min – max	11–37	11–37	19–37
Sex, % male, min – max	27–94	56–90	27–94
Multicenter trials, *n* (%)	23 (46)	18 (58)	5 (26)
*Type of intervention evaluated, n* (%)			
Transfusion or hemostasis therapy	12 (24)	7 (23)	4 (21)
Nutrition	11 (22)	6 (19)	6 (32)
Fluid or blood pressure strategy	7 (14)	4 (13)	3 (16)
Surgical therapy	3 (6)	2 (6)	1 (5)
Imagery	3 (6)	3 (10)	0
Other	14 (28)	9 (29)	5 (26)
Trial presenting with significant results*, n (%)	13 (32)	9 (32)	4 (31)
*Overall ROB, n* (%)			
Low	34 (68)	20 (65)	14 (74)
Some concerns	10 (20)	6 (19)	4 (21)
High	6 (12)	5 (16)	1 (5)

Data are presented as median and interquartile range (IQR), min and max values or number and percentages (%) according to type of variable

ISS: Injury Severity Score. VAP: Ventilator-associated pneumonia. ROB: Risk of bias

^$^Of the 44 trials for which the primary outcome is clearly defined

^*^Of the 41 trials reporting a result for the predefined primary outcome

The number of participants included ranged from 41 to 1629 with a mean number of 233 patients. Included studies demonstrated a wide variation in baseline patient characteristics. The mean age ranged from 31 to 70 years old, and the percentage of male patients ranged from 27 to 94%. Additionally, the ISS ranged from 11 to 37 points, highlighting the variability in the severity of injuries among the studies. Out of the 41 studies that reported a result for the predefined primary outcome, 13 studies (32%) reported statistically significant results.

### Terms & definitions used in RCTs

Among RTCs included, 12 different terms were used. The most frequently used was “*multiple trauma*” that occurred in 42% studies (*n* = 21) [19–39]. The second, “*severe trauma*”, occurred in 16% studies (*n* = 8) [4, 5, 40–45] and was followed by “*major trauma*” [46–49] and “*trauma with hemorrhagic shock*” (8%, n = 4) [3, 50–52]. Other terms used were “*multiple injuries*”[53–55], “*severely injured patients*”[6, 56, 57], “*polytrauma*” [58, 59], “*traumatic hypovolemic shock*” [60], “*seriously injured patients*”[61], “*trauma patients at risk for hemorrhagic shock*”[62], “*multisystem trauma patients*” [63] and “*hypotensive trauma*” [64] **(**Additional file 1: Material 4). The terms were found at least once among the inclusion criteria in XX% of the included RCT (*n* = XX).

**Fig. 2:**
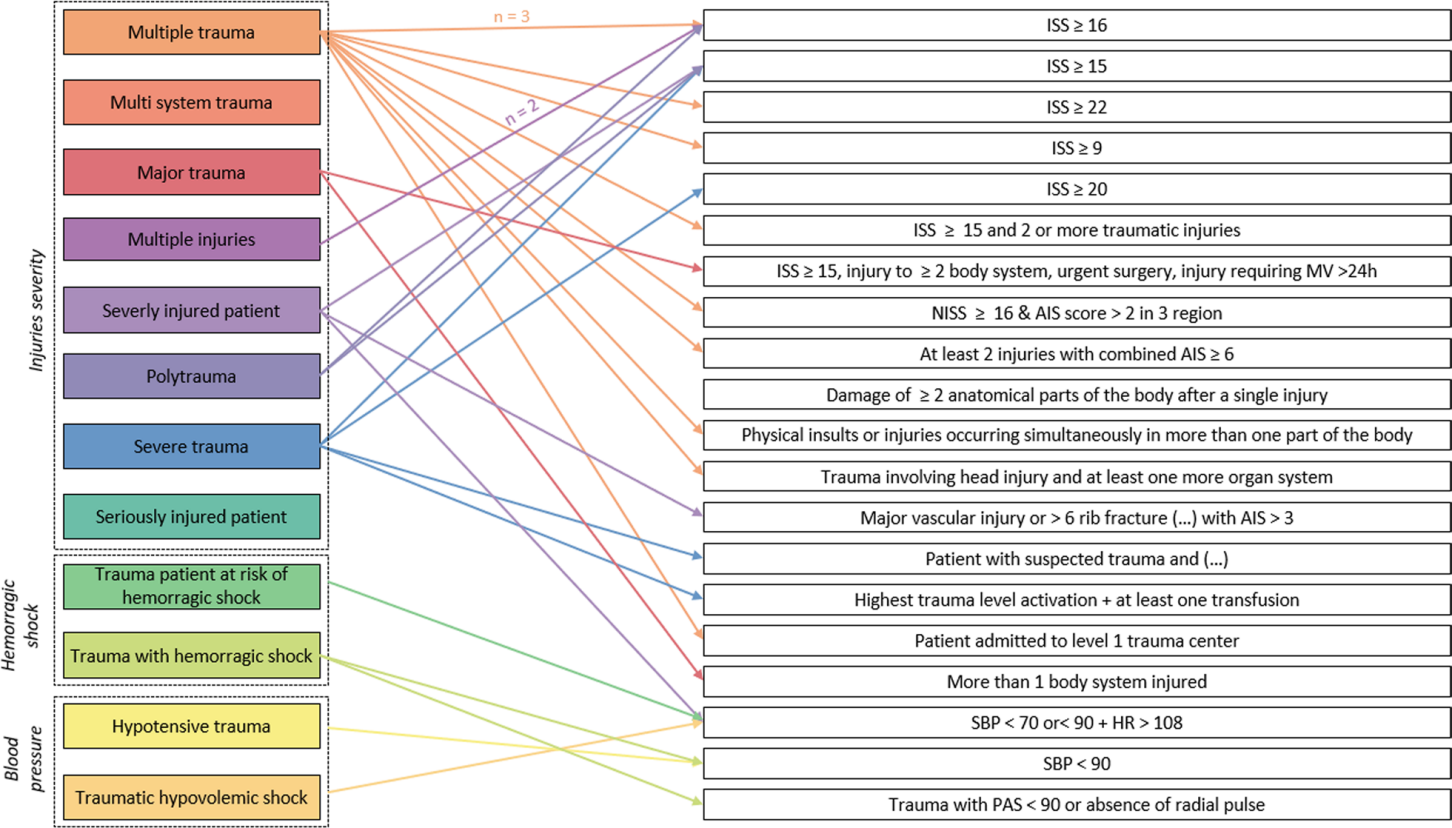
Definitions according to terms used *: major vascular injury or > *6 rib fracture or complex pelvic fracture or* > *20% blood loss or AIS* > *4 for thorax/abdo or* > *3 regions with AIS* > *3. *^*$*^*: Patient with suspected trauma & respiratory rate* > *30/min, pulse* > *120/min, systolic blood pressure* < *100 mmHg, Glasgow coma scale* < *13, estimated exterior blood loss* > *500 mL, abnormal pupillary reaction OR patient with a clinical suspicion of one of the following diagnoses: fractures from at least two long bones, flail chest, open chest or multiple rib fractures, severe abdominal injury, pelvic fracture, unstable vertebral fractures OR fall from a high height, ejection from a vehicle, death of occupant in same vehicle, wedged or trapped chest / abdomen. For clarity, terms used were grouped by similarity in three overarching categories with “injuries severity”, “hemorrhagic shock” and “blood pressure”-related definitions*

Associated with these terms, 21 different definitions were identified. Moreover, 38% of RCTs did not report any definition of the term used (*n* = 19). When a definition was reported, the ISS was the most commonly used criteria (30%, *n *= 15), with a wide range of possible thresholds ranging from 9 to 20, the most frequently used being superior to 15 (16%, *n* = 8). Other definitions included the involvement of at least two body regions in eight studies (16%) or physiological parameters such as systolic blood pressure or heart rate in six studies (12%). Figure 2 reports the broad distribution across terms and related definition, highlighting that a given term can be associated with several definitions, and conversely, that a given definition can be related with several terms. Moreover, among RCTs that reported a definition, only 6% supported the choice with a citation (*n* = 2). Finally, despite the publication of the Berlin definition in 2014, none of the 23 included RCTs published after its release used this definition.

### Impact of the terms on baseline characteristics and outcomes in RCTs

**Fig. 3:**
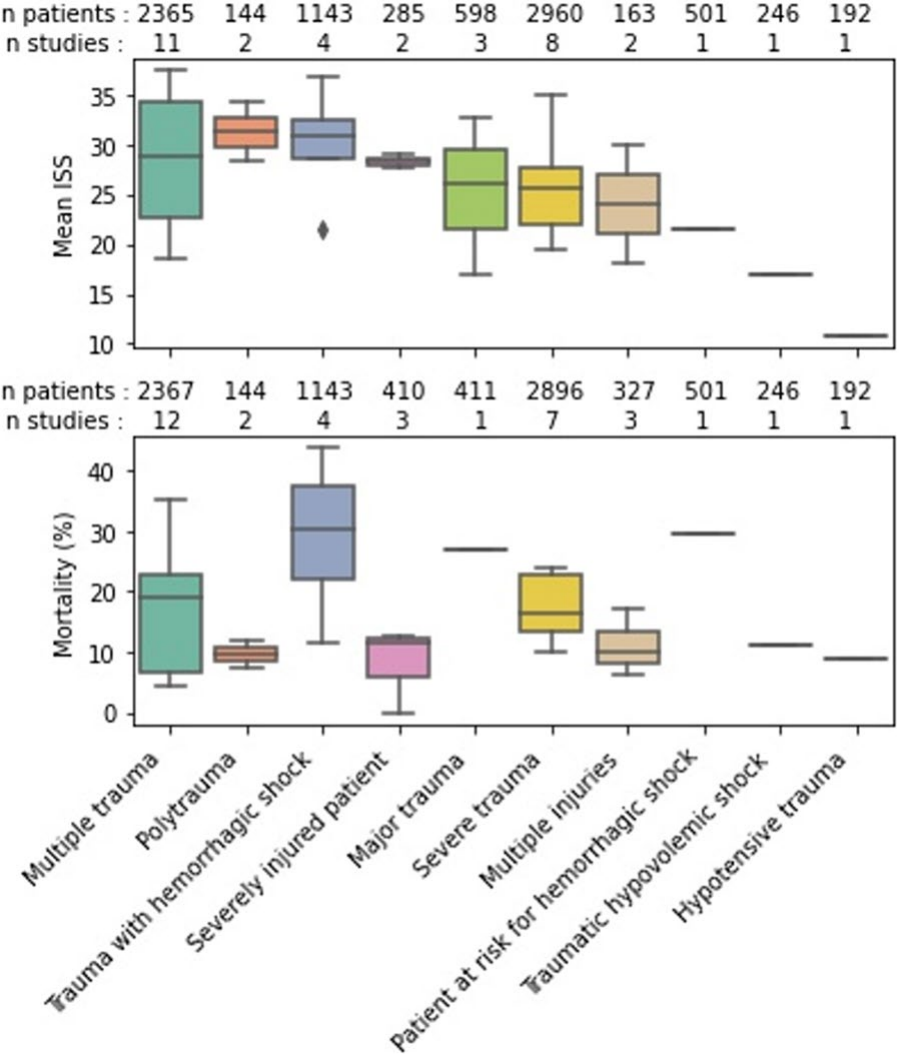
Average ISS and mortality according to terms used. Two terms (Seriously injured patients and Multiple systems trauma patients) are not included in the figure because the studies did not report mortality within these groups.

The choice of terms used did not appear to affect the demographic characteristics of included patients. When grouped by terms used, mean age ranged from 34 to 45, and the percentage of male subjects ranged from 70 to 85%. However, the types and severity of injuries did differ according to the terms chosen. The percentage of included patients suffering from traumatic brain injury ranged from 10 to 84, and the mean ISS ranged from 11 to 32. Furthermore, outcomes varied according to the terms used, as illustrated by the mortality rate, which could range from 9 to 31% (Fig. 3).

### Risk of bias in RCTs

The overall risk of bias was moderate with 68% of studies (34/50) considered at low risk and 12% considered with high risk (6/50) and with some concerns for remaining studies (10/50–20%) (Additional file 1: Material 5). The risk of bias was found to originate from protocol deviation in 12% (*n* = 6/50), from imprecision outcomes measurement in 10% (*n* = 5/50), from selection bias in 10% (*n* = 5/50), from randomization in 8% (*n* = 4/50) and from missing data bias in 6% (*n* = 3/50).

### Terms and definitions used in clinical trial protocols

Among the 11 protocols included, five different terms were used. The most frequently used was “*severe trauma*”, with six occurrences (55%), followed by “*multiple trauma*” (18%, *n* = 2) while other terms were “*severely injured patient*”, “*polytrauma*” and “*multiple injuries*” (9%, *n* = 1). Of these 11 protocols, 45% (*n* = 5) did not provide a definition of the term used.

When a definition was provided, 27% (*n* = 3) relied on the Injury Severity Score (ISS) with thresholds of either > 15 (*n* = 2) or > 18, while other definitions were used only once, such as “*at least one Vittel criteria*”, “*systolic blood pressure inferior to 70 mmHg and heart rate HR superior to 108 bpm*” and “*simultaneous injuries in two or more organs*”.

## Discussion

### Main findings

This systematic review of contemporary RCTs recruiting multiple trauma patients found 12 different terms used to describe this population and that nearly 40% of the RCTs did not report any definition of the term used. Where definitions were included, there were more than 20 variations. These results expose that despite an international consensus in 2014 [13], a substantial heterogeneity remains in the terms and definitions used in RCTs involving multiple trauma patients.

This diversity implies a significant between-trials heterogeneity regarding both baseline characteristics (such as the mean ISS that ranged from 11 to 32) and outcomes (such as mortality ranging between 9 to 31%). The lack of consistency in the terminology used could explain some differences in the required co-interventions, variations in the observed treatment effects and finally the low rate of significant outcomes observed in our study and in the existing literature.

### Discussion with existing literature

In 2009, a systematic review including any type of studies identified 47 possible definitions of multiple trauma [12]. Almost fifteen years later, our review underpins the persistence of this heterogeneity both in the terms used and, in the definition associated with these terms. This issue of variability in defining a specific clinical conditions for trials has been previously highlighted in various contexts, including ARDS [65], traumatic hemorrhagic shock [66] or refractory septic shock [67].

The time-sensitive nature of trauma care leads to the challenge of specifying a consistent and unique definition. As an example, the selection of variables used in the definition involves a balance between the availability of variables and the timing of the intervention being evaluated. For instance, variables such as AIS or ISS are strongly associated to patient severity, making them suitable for inclusion in a definition, as proposed by the Pape et al. [13]. However, these variables rely on the completion and interpretation of a full-body CT scan which limits the use of this definition for research projects carried out prior to scanning. Furthermore, it restricts the use of this definition in low-income settings with limited resources such as CT scans. On the other hand, some variables may be available very early after the trauma, such as blood pressure or heart rate [3, 62], but these variables, if only considered at one time point are also likely to be less specific, potentially failing to include the population of interest.

Nevertheless, standardized consistent definitions are possible. Time-sensitive conditions have been defined as demonstrated with the Berlin definition for ARDS [68] or SEPSIS-III definition [69]. The strengths of these two definitions rely on objective, easily measurable and accurate clinical criteria that can be promptly measured and capture essential criteria of each syndrome. These characteristics allow for simple use and offer consistency, whether applied prospectively or retrospectively.

### Limitations

First, defining the scope of a systematic review involves defining a population. This was a methodological challenge as analyzing this definition was the main aim of this systematic review. Thus, we chose to use a spectrum of synonyms of multiple trauma in the search equation and to include after titles and abstracts screening those RCTs that reported authors commitment to include trauma with a certain severity. This choice was guided by systematic reviews that have had a similar focus in other clinical situations such as polypharmacy [70], community health workers [71] or labor [72]. It nevertheless leads to the exclusion of RCTs such as CRASH II that reported in their abstract the intention to include “*adult trauma patients with, or at risk of, significant bleeding*” and that are not indexed in PubMed under the Mesh “*multiple trauma*” [73].

Second, due to the broad diversity of populations, interventions and outcomes included in this systematic review, it was not possible to evaluate the impact of the choice of terms or definition on the effectiveness of the interventions. This study only reports that providing a clear definition of the term used did not seems to be associated with an increased proportion of significantly positive primary outcomes.

Third, there is a possibility that some terms or definitions may have been missed, especially if the trial that used them was not within the scope of the systematic review. As a result, an additional term or definition could emerge, or another occurrence of a term or definition already included in the systematic review. Such an event would not alter the message of the review but would only emphasize the importance of the existing heterogeneity.

Fourth, this study exclusively focuses on the terms employed to denote the, though it can be argued that the true determinant of the recruited population lies within inclusion criteria. Such a statement might downplay the importance of the heterogeneity exposed in this study. Nonetheless, our findings also expose that terms such as “multiple trauma” or “severe trauma” are frequently used within the included manuscripts inclusion criteria section. Such utilization overall strengthens the problematic exposed as all these terms convey a certain degree of ambiguity. Indeed, even if widely acknowledged that, irrespective of the specific term employed, these patients are at a heightened risk of poor outcomes, an incredible diversity of potential clinical presentations exists, and this diversity can ultimately lead to the categorization of markedly distinct patients under the same generic overarching term.

Finally, it is not possible, within the context of this work, to recommend the use of one term or definition over another. The sole aim of this systematic review was to determine which terms were commonly used in the literature and which definitions were associated with these terms. Therefore, the purpose of this work was not to identify a consensus definition or to determine whether a given definition was more often associated with a significant outcome.

### Implications

Also, the lack of consistency in definitions and underlying clinical heterogeneity presents a challenge for integrating previously published evidence. Meta-analysis assumes that populations are similar enough to be pooled into a single measure of effect, but this assumption is undermined when authors fail to provide a clear (or any) definition. This incomplete reporting has been shown to significantly contribute to research waste [74].

The observed heterogeneity in definitions may also contribute to physicians' uncertainty at the bedside. In a prospective observational study, trauma surgeons have been reported to only reach a moderate agreement regarding whether a given patient should be qualified as a multiple trauma or not [75]. This finding challenges the common belief that caregivers base their health care diagnosis on rigorous definitions and emphasize the need of a standardization of these which encompass the complexity and time-sensitive nature of trauma care.

The heterogeneity in definitions used may also reflect the presence of several phenotypes within this population, as it has been advocated for ARDS [76]. It could indeed be argued that severe traumatic brain injury and hemorrhagic shock, as well as penetrating and non-penetrating trauma, are different diseases. In this light, it might be necessary to consensually delineate subgroups within the definition to acknowledge for these differences [77].

Finally, for stakeholders involved in the design of future RTCs, it may be important to acknowledge that the terms used can have a direct impact on critical outcomes, such as the mortality rate. This awareness can be particularly relevant when determining the appropriate number of patients to treat.

## Conclusion

In conclusion, our systematic review has revealed significant heterogeneity in the terms and in definitions used to qualify multiple trauma patients in randomized controlled trials. This underscores the importance of further efforts to establish a unique and consistent definition of multiple trauma, taking into consideration the time-sensitive nature of this pathology.

### Supplementary Information

**Additional file 1.** **Supplementary Material 1:** Check list PRISMA. **Supplementary Material 2:** Research Algorithm for RCT & Protocols. **Supplementary Material 3:** Characteristics of included studies. **Supplementary Material 4:** Distribution of terms used. **Supplementary Material 5:** Risk of bias assessment.

## Data Availability

All data used during the current study are available from the corresponding author upon reasonable request.

## Abbreviations

ISS: Injury Severity Score
RCT: Randomized Controlled Trial
ARDS: Acute Respiratory Distress Syndrome
AIS: Abbreviated Injury Scale
HLA-DR: Human Leukocyte Antigen-DR Isotype
CT: Computed Tomography scan
