# Supplementary material for: Heterogeneity in defining multiple trauma: a systematic review of randomized controlled trials
Source: Crit Care. 2023 Sep 22;27:363. doi: 10.1186/s13054-023-04637-w (PMC10515068; doi:10.1186/s13054-023-04637-w)

# Supplementary materials

## Supplementary Material 1: Check list PRISMA

| **Section and Topic** | **Item #** | **Checklist item** | **Location where item is reported** |
| --- | --- | --- | --- |
| **TITLE** | | |  |
| Title | 1 | Identify the report as a systematic review. | Title |
| **ABSTRACT** | | |  |
| Abstract | 2 | See the PRISMA 2020 for Abstracts checklist. | Appendix 1 |
| **INTRODUCTION** | | |  |
| Rationale | 3 | Describe the rationale for the review in the context of existing knowledge. | Intro |
| Objectives | 4 | Provide an explicit statement of the objective(s) or question(s) the review addresses. | Intro |
| **METHODS** | | |  |
| Eligibility criteria | 5 | Specify the inclusion and exclusion criteria for the review and how studies were grouped for the syntheses. | Inclusion & Exclusion criteria |
| Information sources | 6 | Specify all databases, registers, websites, organisations, reference lists and other sources searched or consulted to identify studies. Specify the date when each source was last searched or consulted. | Data source & strategy |
| Search strategy | 7 | Present the full search strategies for all databases, registers and websites, including any filters and limits used. | Appendix 2 |
| Selection process | 8 | Specify the methods used to decide whether a study met the inclusion criteria of the review, including how many reviewers screened each record and each report retrieved, whether they worked independently, and if applicable, details of automation tools used in the process. | Selection of studies |
| Data collection process | 9 | Specify the methods used to collect data from reports, including how many reviewers collected data from each report, whether they worked independently, any processes for obtaining or confirming data from study investigators, and if applicable, details of automation tools used in the process. | Data extraction |
| Data items | 10a | List and define all outcomes for which data were sought. Specify whether all results that were compatible with each outcome domain in each study were sought (e.g. for all measures, time points, analyses), and if not, the methods used to decide which results to collect. | Data extraction |
|  | 10b | List and define all other variables for which data were sought (e.g. participant and intervention characteristics, funding sources). Describe any assumptions made about any missing or unclear information. | Statistical Analysis |
| Study risk of bias assessment | 11 | Specify the methods used to assess risk of bias in the included studies, including details of the tool(s) used, how many reviewers assessed each study and whether they worked independently, and if applicable, details of automation tools used in the process. | Risk of bias assessment |
| Effect measures | 12 | Specify for each outcome the effect measure(s) (e.g. risk ratio, mean difference) used in the synthesis or presentation of results. | Statistical Analysis |
| Synthesis methods | 13a | Describe the processes used to decide which studies were eligible for each synthesis (e.g. tabulating the study intervention characteristics and comparing against the planned groups for each synthesis (item #5)). | Data extraction |
|  | 13b | Describe any methods required to prepare the data for presentation or synthesis, such as handling of missing summary statistics, or data conversions. | Statistical Analysis |
|  | 13c | Describe any methods used to tabulate or visually display results of individual studies and syntheses. | Statistical Analysis |
|  | 13d | Describe any methods used to synthesize results and provide a rationale for the choice(s). If meta-analysis was performed, describe the model(s), method(s) to identify the presence and extent of statistical heterogeneity, and software package(s) used. | No meta-analysis |
|  | 13e | Describe any methods used to explore possible causes of heterogeneity among study results (e.g. subgroup analysis, meta-regression). | No sub groups analysis |
|  | 13f | Describe any sensitivity analyses conducted to assess robustness of the synthesized results. | No sensitive analyse |
| Reporting bias assessment | 14 | Describe any methods used to assess risk of bias due to missing results in a synthesis (arising from reporting biases). | Risk of bias assessment |
| Certainty assessment | 15 | Describe any methods used to assess certainty (or confidence) in the body of evidence for an outcome. | No certainty assessment |
| **RESULTS** | | |  |
| Study selection | 16a | Describe the results of the search and selection process, from the number of records identified in the search to the number of studies included in the review, ideally using a flow diagram. | Figure 1 |
|  | 16b | Cite studies that might appear to meet the inclusion criteria, but which were excluded, and explain why they were excluded. | Search results |
| Study characteristics | 17 | Cite each included study and present its characteristics. | Appendix 3 |
| Risk of bias in studies | 18 | Present assessments of risk of bias for each included study. | Appendix 5 |
| Results of individual studies | 19 | For all outcomes, present, for each study: (a) summary statistics for each group (where appropriate) and (b) an effect estimate and its precision (e.g. confidence/credible interval), ideally using structured tables or plots. | Figure 2 & Appendix 4 |
| Results of syntheses | 20a | For each synthesis, briefly summarise the characteristics and risk of bias among contributing studies. | Risk of bias |
|  | 20b | Present results of all statistical syntheses conducted. If meta-analysis was done, present for each the summary estimate and its precision (e.g. confidence/credible interval) and measures of statistical heterogeneity. If comparing groups, describe the direction of the effect. | No statistical test |
|  | 20c | Present results of all investigations of possible causes of heterogeneity among study results. | 0 |
|  | 20d | Present results of all sensitivity analyses conducted to assess the robustness of the synthesized results. | 0 |
| Reporting biases | 21 | Present assessments of risk of bias due to missing results (arising from reporting biases) for each synthesis assessed. | 0 |
| Certainty of evidence | 22 | Present assessments of certainty (or confidence) in the body of evidence for each outcome assessed. | 0 |
| **DISCUSSION** | | |  |
| Discussion | 23a | Provide a general interpretation of the results in the context of other evidence. | Discussion with ongoing litterature |
|  | 23b | Discuss any limitations of the evidence included in the review. | Limitations |
|  | 23c | Discuss any limitations of the review processes used. | Limitations |
|  | 23d | Discuss implications of the results for practice, policy, and future research. | Implications |
| **OTHER INFORMATION** | | |  |
| Registration and protocol | 24a | Provide registration information for the review, including register name and registration number, or state that the review was not registered. | Not registered |
|  | 24b | Indicate where the review protocol can be accessed, or state that a protocol was not prepared. | 0 |
|  | 24c | Describe and explain any amendments to information provided at registration or in the protocol. | 0 |
| Support | 25 | Describe sources of financial or non-financial support for the review, and the role of the funders or sponsors in the review. | No sources |
| Competing interests | 26 | Declare any competing interests of review authors. | Conlicts of interest |
| Availability of data, code and other materials | 27 | Report which of the following are publicly available and where they can be found: template data collection forms; data extracted from included studies; data used for all analyses; analytic code; any other materials used in the review. | Data availibility |

## Supplementary Material 2: Research Algorithm for RCT & Protocols

**PubMed**: (Date of completion: 04/08/2022)

#Multiple trauma#

1. severe trauma[tiab]

2. severe injur*[tiab]

3. poly trauma [tiab]

4. polytrauma[tiab]

5. multiple wound*[tiab]

6. multiple injur*[tiab]

7. multiple trauma[tiab]

8. major trauma[tiab]

9. major injur*[tiab]

10. multiple trauma [MeSH Terms]

11. #1 OR #2 OR #3 OR #4 OR #5 OR #6 OR #7 OR #8 OR #9 OR #10

#Randomized Study#

12. “randomized controlled trial”[pt]

13. “controlled clinical trial”[pt]

14. randomized[tiab]

15. placebo[tiab]

16. “drug therapy”[sh]

17. randomly[tiab]

18. trial[tiab]

19. groups[tiab]

20. #12 OR #13 OR #14 OR #15 OR #16 OR #17 OR #18 OR #19

21. animals[mh] NOT humans[mh]

22. #20 NOT #21

#Adults#

23. adult[mh] NOT child[mh] NOT adolescent [mh] NOT infant[mh]

#2002 – 2022#

24. "2002/01/01"[Date - Publication] : "2022/08/01"[Date - Publication]

#Total#

25. #11 AND #22 AND #23 AND #24

((((((((((((severe trauma[tiab]) OR (severe injur*[tiab])) OR (poly trauma [tiab])) OR (polytrauma[tiab])) OR (multiple wound*[tiab])) OR (multiple injur*[tiab])) OR (multiple trauma[tiab])) OR (major trauma[tiab])) OR (major injur*[tiab])) OR (multiple trauma [MeSH Terms])) AND ((((((((("randomized controlled trial"[pt]) OR ("controlled clinical trial"[pt])) OR (randomized[tiab])) OR (placebo[tiab])) OR ("drug therapy"[sh])) OR (randomly[tiab])) OR (trial[tiab])) OR (groups[tiab])) NOT (animals[mh] NOT humans[mh]))) AND (adult[mh] NOT child[mh] NOT adolescent [mh] NOT infant[mh])) AND ("2002/01/01"[Date - Publication] : "2022/08/01"[Date - Publication])

**Embase**: (Date of completion: 04/08/2022)

#Multiple trauma#

1. (“severe trauma”):ab,ti

2. (severe NEXT/1 injur*):ab,ti

3. (“poly trauma”):ab,ti

4. (polytrauma):ab,ti

5. (multiple NEXT/1 wound*):ab,ti

6. (multiple NEXT/1 injur*):ab,ti

7. (“multiple trauma”):ab,ti

8. (“major trauma”):ab,ti

9. (major NEXT/1 injur*):ab,ti

10. (“Multiple trauma”)/exp

11. #1 OR #2 OR #3 OR #4 OR #5 OR #6 OR #7 OR #8 OR #9 OR #10

#Adults – 2002/2022#

12. [humans]/lim AND [english]/lim AND [1-1-2002]/sd NOT [1-08-2022]/sd AND [adult]/lim

13. #11 AND #12

#Randomized study#

14. #13 AND (randomized controlled trial'/de)

#Embase#
15. #14 AND [embase]/lim

**ClinicalTrial** (Date of completion: 04/08/2022)

Interventional Studies | severe trauma OR major trauma OR multiple trauma OR polytrauma OR multiple injur* | Adult, Older Adult | Phase 3

## Supplementary Material 3: Characteristics of included studies

| **Terms used** | **First author**  **Year**  **DOI** | **Inclusion criteria** | **Intervention** | **Control** | **Number of patients included** | **Main outcome** | **Definition** |
| --- | --- | --- | --- | --- | --- | --- | --- |
| Multiple trauma | Tsilika, M  2022  10.1016/j.ijantimicag.2021.106471 | Recent trauma involving head injury and at least one more system, intubation immediately after injury, with expected duration of ventilation > 10 day | Combination of four probiotics | Placebo | 112 | VAP within the first 30 days | Trauma involving head injury and at least one more organ system |
|  | Zhang, X  2022  10.1155/2022/8929418 | Multiple trauma expected to be hospitalized more than 1 week | Comprehensive nursing | Routine care | 68 | Self-made quality of life score sheet | Simultaneous or sequential damage of two or more anatomical parts of the body |
|  | Kagan, I  2020  10.1002/jpen.2025 | Multiple trauma patient who require mechanical ventilation | Diet with fish oil | Diet with omega 6 PUFA | 51 | Not specified | No definition |
|  | Habib, T  2020  10.22159/ajpcr.2020.v13i10.38114 | Multiple trauma patients on mechanical ventilator | Probiotics | Placebo | 65 | VAP | No definition |
|  | Akbari, E  2018  10.1016/j.ajem.2018.02.018 | Severe blunt multiple Trauma in need of transfusion and with Fg < 200 mg/dL | Group 1: 2g of Fibrinogen –  Group 2: 2 FFP | No product other than RBC | 90 | Mortality | ISS > 16 |
|  | Kahn, J  2016  10.1177/0284185115580839 | Preclinically and clinically suspected multiple trauma | Scan with adaptive statistical iterative reconstruction | Scan using a FBP protocol | 122 | Not specified | ISS ≥ 16 |
|  | Grintescu, IM  2015  10.1016/j.clnu.2014.05.006 | ISS 22 or more, and a requirement for early parenteral nutritional | Parenteral supplementation with N(2)-Lalanyl-L-glutamine dipeptide ( | Standard amino acid solution | 82 | Blood glucose level | ISS ≥ 22 |
|  | Kagan, I  2015  10.1007/s00134-015-3646-z | Multiple trauma who required mechanical ventilation | Enteral nutrition enriched with supplemental EPA, GLA & antioxidants | Enteral nutrition alone | 120 | PaO2/FiO2 at day 4 & 8 | Physical insults or injuries occurring simultaneously in more than one part of the body |
|  | Curry, N  2015  10.1093/bja/aev134 | Adult trauma patients with actively bleeding and required activation of the MHP | Two early pools of cryoprecipitate, given within 90 min of admission | Major haemorrhage therapy alone | 43 | percentage of subjects randomized to the intervention (CRYO) arm in receipt of cryoprecipitate within 90 min | No definition |
|  | Roquilly, A  2011  10.1001/jama.2011.360 | Patients with multiple trauma who were older than 15 years 3 months and expected to require mechanical ventilation for more than 48 hours | HSC | NaCl | 149 | VAP at d28 | 2 or more traumatic injuries and an injury severity score higher than 15 |
|  | Arefian, NM  2007  -^1^ | Multiple trauma patient with GCS 4-10 at the end of the first day | Partial parenteral nutrition | Enteral nutrition | 80 | Not specified | No definition |
|  | Pirente, N  2007  10.1007/s00423-007-0171-8 | At least two injuries with a combined AIS ≥6, age between 18 and 70 years, and mental orientation | Standard care + standardised psychotherapy | standard care | 130 | HRQOL at 6 months | At least two injuries with a combined abbreviated injury scale (AIS) severity index ≥6 |
|  | Stoutenbeek, CP  2006  10.1007/s00134-006-0455-4 | Patients admitted to ICU after non penetrating trauma | Selective decontamination 4 times a day | Standard care | 401 | Mortality in ICU | ISS ≥ 16 |
|  | Velmahos, GC  2005  10.1016/j.surg.2005.01.010 | Trauma patient with contraindication to anticoagulant | Electrostimulation | Standard care | 47 | Deep venous thrombosis | ISS > 9 |
|  | Chytra I  2007  10.1186/cc5703 | Ventilated patients with multiple trauma and estimated blood loss of more than 2,000 ml | Esophageal doppler guided fluid management | No doppler | 162 | Blood lactate levels 12 and 24 hours after ICU admission and organ dysfunction development during ICU stay | No definition |
|  | Cotae, AM  2021  10.3390/medicina57050408 | Trauma patient undergoing emergency surgery expected to last at least 2h | Entropy-guided anesthesia | No entropy | 95 | Post operative cognitive dysfunction | No definition |
|  | Najafi, A  2014  10.1186/2008-2231-22-57 | Multiple traumas with mechanical ventilation with FiO2 more than 50%, APACHE II > 15, suspected infection and at least two SIRS criteria | Standard treatment + NAC | Standard treatment | 41 | HBD2 levels | No definition |
|  | Saltzherr TP  2012  10.1002/bjs.7705 | All injured patients who fulfilled the prehospital triage criteria for transport to a level 1 trauma center | CT in the trauma room | CT outside the trauma room | 1045 | Number of non-institutionalized days during the first year after the trauma | Patient admitted to level 1 trauma center |
|  | MTCTG  2005  -^2^ | All trauma patients admitted to Trauma ICU | Gastric pHi-driven rescucitation protocol | Routine trauma after placement of a gastric tonometer | 151 | ICU mortality | No definition |
|  | Pape, HC  2007  10.1097/SLA.0b013e3181485750 | Multiple injuries with a New ISS exceeding 16 points or 3 AIS > 2 + a long bone midshaft fracture of the lower extremity | Intramedullary femoral nailing | Eternal fixation, followed by secondary intramedullary nailing | 165 | Incidence of pulmonary complications | NISS > 16 or AIS score > 2 in 3 regions |
|  | Pneumatikos, I  2002  10.1007/s00134-002-1238-1 | Multiple traumas admitted to ICU with mechanical ventilation and an expected time of 5 days with mechanical ventilation | Selective decontamination in the subglottic area | Placebo | 61 | Cumulative VAP | No definition |
| Severe Trauma | Marjanovic, N  2021  10.1016/j.chest.2021.03.007 | severe trauma requiring mechanical ventilation | Automatic control of the tracheal tube’s cuff pressure | Manual control of the tracheal tube’s cuff pressure | 434 | proportion of patient developping VAP during first 28d | ISS > 15 |
|  | Sierink, JC 2016  10.1016/S0140-6736(16)30932-1 | Patients aged 18 years or older with compromised vital parameters, clinical suspicion of life-threatening injuries, or severe injury | Immediate total-body CT scanning without previous conventional imaging | Standard work up | 1403 | In hospital mortality | Patient with suspected trauma & RR >30, FC >120, PAS < 100, GCS < 13, estimated exterior blood loss > 500 mL, abnormal pupillary reaction. OR patient with a clinical suspicion of one of the following diagnoses: fractures from at least two long bones, flail chest, open chest or multiple rib fractures, severe abdominal injury, pelvic fracture, unstable vertebral fractures OR fall from a height, ejection from a vehicle, death of occupant in same vehicle, wedged or trapped chest / abdomen |
|  | Holcomb, JB  2015  [10.1001/jama.2015.12](https://pubmed.ncbi.nlm.nih.gov/?term=Holcomb+JB&cauthor_id=25647203) | Highest level trauma activation + Initiated transfusion of at least 1 U of blood component within the first hour of arrival or during prehospital transport | 1:1:1 transfusion ratio | 1:1:2 transfusion ratio | 680 | Absolute percentage group differences for 24-hour and 30- day mortality | Highest trauma level activation + at least one transfusion during first hour |
|  | James, MFM  2011  10.1093/bja/aer229 | Penetrating and blunt trauma requiring > 3 L volume resuscitation | HES 130/0.4 | Saline 0,9 | 115 | Volume of FIRST fluid needed in the first 24h after enrolment | No definition |
|  | Nascimento, B  2016  10.1093/bja/aew343 | Severe trauma at risk of significant hemorrhage | 6g fibrinogen concentrate | Saline serum | 45 | Feasibility: proportion of subject receiving intervention | No definition |
|  | Nascimento, B  2013  10.1503/cmaj.121986 | Patients with traumatic injurie, who had bleeding, were expected to receive massive transfusion, and an episode of SBP < 90 | Transfusion in 1:1:1 ratio | Standard care | 69 | Feasibility: proportion of subject receiving intervention | No definition |
|  | Zhao, XD  2013  10.1007/s11596-013-1080-4 | ISS > 20 and blood glucose test after admission > 9 at 3 times | Intensive insulin therapy group | Standard treatment | 64 | ND | ISS ≥ 20 |
|  | Spinella, PC  2020  10.3389/fimmu.2020.02085 | Traumatic injury which requires at least one transfusion or one emergent operation | Group 1: 2g TXA  Group 2 : 4g TXA | placebo | 150 | Reduction in HLA-DR after 72h | No definition |
| Major Trauma | Holmes, A  2007  10.1080/00048670701634945 | All patients admitted to the two level 1 trauma centers in Melbourne | Interpersonal counselling therapy | Standard care | 90 | Prevalence of major depression, PTSD, alcohol or substance use | ISS > 15, serious injury to two or more body system, urgent surgery for non-limb injuries, injuries requiring mechanical ventilation for > 24h |
|  | Baksaas‑Aasen, K  2021  10.1007/s00134-020-06266-1 | Trauma patient for whom MHP have been activated | VHA assays use | Standard care | 411 | Proportion of patient at 24h alive & free of massive transfusion | No definition |
|  | Costa, ML  2020  10.1001/jama.2020.0059 | Patients aged > 16 with lower extremities fracture caused by major trauma | Incisional negative pressure wound therapy | Standard wound dressing | 1629 | Deep surgical site infection | More than 1 body system is injured |
|  | Helm, M  2003  10.1093/bja/aeg069 | Trauma patient treated before admission by intubation | Monitoring capnography | Monitoring blinding | 97 | Incidence of "normal" PaCO2 | No definition |
| Trauma with hemorrhagic shock | Hauser, CJ  2011  10.1097/TA.0b013e3181edf36e | Patients with active hemorrhage caused by trauma who had already received 4 units of red blood cells | rFVIIa | placebo | 573 | 30-day mortality | No definition |
|  | Chrombie, N  2022  10.1016/S2352-3026(22)00040-0 | Traumatic injury with hypotension (SBP < 90) | packed red blood cells (PRBC) and plasma | placebo | 432 | mortality before discharge of ICU or failure to clear lactates | trauma with PAS < 90 or absence of radial pulse |
|  | Morrison, CA  2011  10.1097/TA.0b013e31820e77ea | Traumatic injury requiring emergent laparotomy or thoracotomy + at least one documented SBP < 90 + patient thought to be in hemorrhagic shock by surgeon's judgment | Target MAP 50 mmHg | Target MAP 65 mmHg | 90 | 30 days mortality | SBP < 90 |
|  | Curry, N  2018  10.1186/s13054-018-2086-x | Trauma patients actively bleeding and in hemorrhagic shock and therefore required activation of the MHP | Fibrinogen concentrate | Placebo | 48 | Feasibility: proportion of subject receiving intervention | No definition |
| Multiples injuries | Spindler-Vesel, A  2007  10.1177/0148607107031002119 | Multiple injured patients with at least 4 days of ICU stay | Group A: glutamine;  Group B: fermentable fiber;  Group C: peptide diet  Group D: standard enteral formula with fibers combined with Synbiotic 2000 | | 113 | Lactulose/mannitol excretion | ISS > 18 |
|  | Ma, J  2021  -^3^ | Severe multiple injuries and traumatic shock | Sodium bicarbonated ringer solution | Sodium ringer lactate solution | 50 | coagulation function before and after resuscitation + levels of acids lactics and pH | No definition |
|  | Lu, Y  2018  10.12669/pjms.345.15465 | Admitted to ICU because of severe multiple injuries, ISS > 16, with hemorrhagic shock, MAP < 65 mmHg or systolic pressure lower than 40 mmHg, and have undergone hemostatic treatment one or two hours after admission | MAP 40-50 | MAP 60-80 | 164 | Not reported | ISS > 16 |
| Severely injured patient | Bible, LE  2014  10.1097/TA.0000000000000264 | Major vascular injury or > 6 rib fracture or complex pelvic fracture or > 20% blood loss or AIS > 4 for thorax/abdo or > 3 regions with AIS > 3 | HR reduction with propranolol during 30 days | Standard care | 45 | HPC mobilization, persistent elevation of G-CSF, and anemia following severe injury | Major vascular injury or > 6 rib fracture or complex pelvic fracture or > 20% blood loss or AIS > 4 for thorax/abdo or > 3 regions with AIS > 3 |
|  | Moore, HB  2018  10.1016/S0140-6736(18)31553-8 | Injured adults with SBP < 70 mmHg or 71–90 mmHg and HR > 108 thought to be due to acute blood loss | Two units of AB plasma | Normal saline | 125 | 28 days mortality | Systolic blood pressure (SBP) 70 mm Hg or lower or 71–90 mm Hg and heart rate 108 beats per min thought to be due to acute blood loss |
|  | Ho, KM  2019  10.1056/NEJMoa1806515 | ISS > 15 & contraindication to receipt prophylactic anticoagulation | Vena cava filter | No cava filter | 240 | Symptomatic pulmonary embolism or death at day 90 | ISS > 15 |
| Polytrauma | Liu, T  2019  10.1016/j.jss.2019.04.005 | Polytrauma with ISS > 16 and flail chest | Surgical treatment of flail chest | Conservative treatments | 50 | Mechanical ventilation duration | ISS ≥ 16 |
|  | Innerhofer, P  2017  10.1016/S2352-3026(17)30077-7 | ISS > 15 & clinical sign or risk of significant hemorrhage | Fibrinogen or CFC | FFP | 94 | SOFA during ICU stay | ISS > 15 |
| Traumatic Hypovolemic Shock | Han, J  2015  10.1097/SHK.0000000000000303 | Trauma victims with a SBP < 70 mmHg or 70 to 90 mmHg and a HR ≥ 108 | Hypertonic serum | Ringer Lactates | 246 | ND | SBP < 70 or < 90 + HR > 108 |
| Multi system trauma patient | Boelens PG  2002  10.1093/jn/132.9.2580 | Patients between 18 and 65 with an expected survival of 48h, an ISS of 20 and at least 5d of enteral nutrition | Glutamine supplemented enteral nutrition | Balanced enteral nutrition | 80 | Expression of HLA DR | ISS ≥ 20 |
| Hypotensive Trauma | Schreiber, MA  2015  10.1097/TA.0000000000000600 | Blunt or penetrating trauma, out of hospital SBP < 90, absence of evidence of a severe head injury | Restrictive strategy with fluid during prehospital | liberal strategy | 192 | 24h mortality | SBP < 90 |
| Seriously injured patients | Khan, FA  2016  10.1007/s00068-015-0563-3 | Seriously injured patients with hemorrhagic shock (SBP < 80) and obvious need for operative intervention | 1g of Methylprednisolone | Placebo | 118 | Incidence of reduced renal perfusion and function | No definition |
| Trauma Patients at Risk for Hemorrhagic Shock | Sperry, JL  2018  10.1056/NEJMoa1802345 | Patient transported to a trauma center with at least an episode of SBP < 90 and HR > 108 or SBP < 70 | Administration of 2 units of thawed plasma during air transport | Standard care | 501 | 30 days mortality | At least an episode of SBP < 90 and HR > 108 or SBP < 70 |

*VAP : Ventilator associated pneumoniae ; ICU : Intensive Care Unit ; SBP : Systolic Blood Pressure ; ISS : Injury Severity Score ; MHP : Major Haemorraghic Procedure ; VHA : Viscoelastic haemostasis assays ; TXA : Tranexamic Acid ; Fg : Fibrinogen ; FFP : Fresh Frozen Plasma ; RBC : Red Blood Cells ; MAP : Mean Arterial Pressure ; CFC : coagulation factors concentrate ; CT : Computed Tomography ; SOFA : Sepsis related Organ Failure Assessment ; FDB : filtered back projection ; EPA : eicosapentaenoic acid ; GLA : gamma-linolenic acid ; HSC : Hydrocortisone Hemisuccinate ; rVIIA : activated factor VII ; GCS : Glasgow Cerebral Score ; AIS : Abbrievated Injury Score ; HRQOL : Health Related Quality Of Life ; HR : Heart Rate ; HPC : hematopoietic progenitor cells ;*

1. *Arefian NM, Teymourian H.Effect of partial parenteral versus enteral nutritional therapy on serum indices in multiple trauma patients. Tanaffos. 2007.*
2. *Miami Trauma Clinical Trials Group. Splanchnic hypoperfusion-directed therapies in trauma: a prospective, randomized trial. Am Surg. 2005 Mar;71(3):252-60. PMID: 15869144.*
3. *Ma J, Han S, Liu X, Zhou Z. Sodium bicarbonated Ringer's solution effectively improves coagulation function and lactic acid metabolism in patients with severe multiple injuries and traumatic shock. Am J Transl Res. 2021 May 15;13(5):5043-5050. PMID: 34150090; PMCID: PMC8205763.*

##
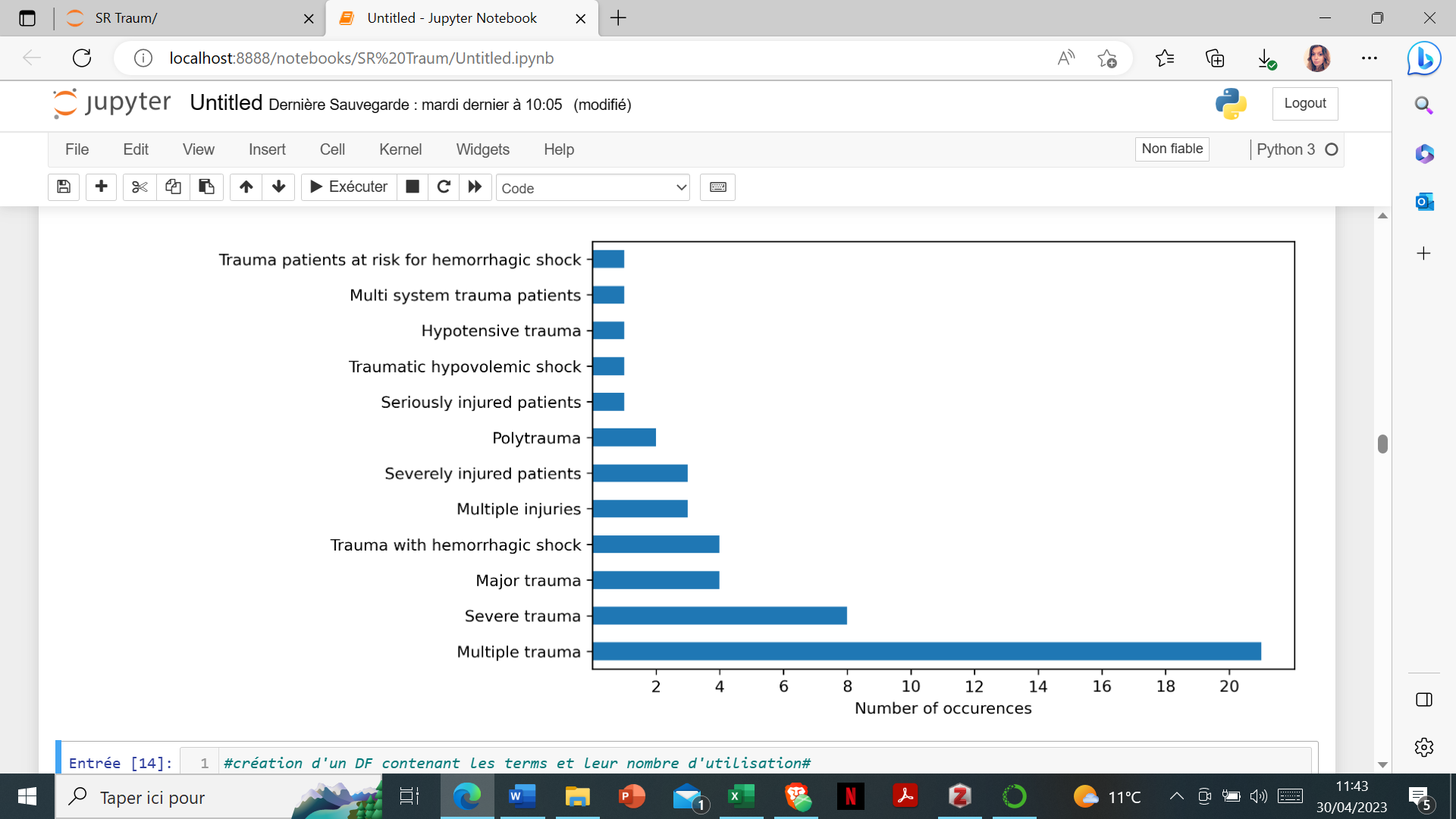
 Supplementary Material 4: Distribution of terms used

## Supplementary Material 5: Risk of bias assessment


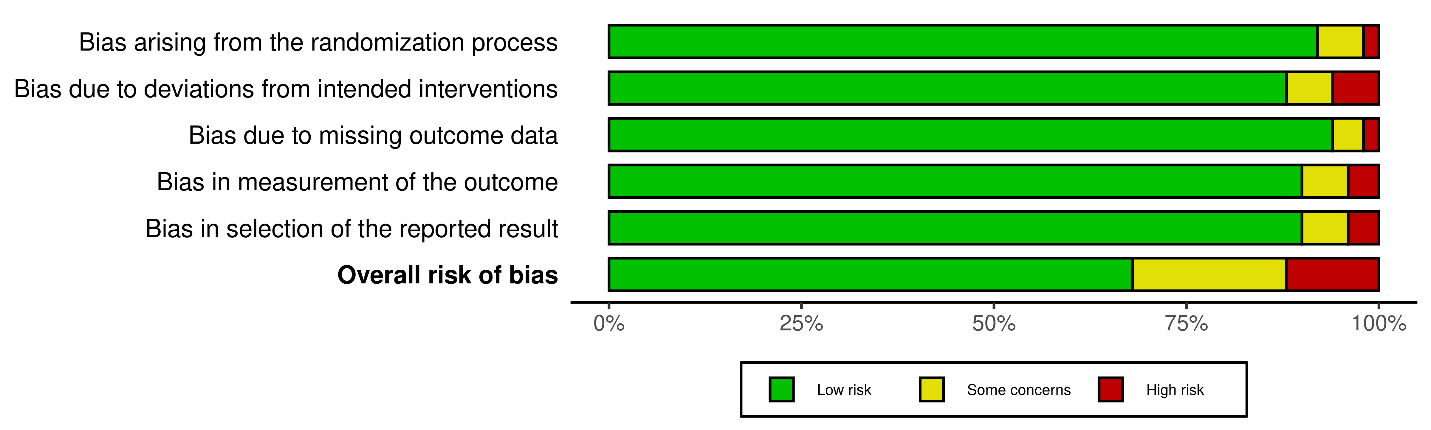

Supplement: Supplementary file 1 — Additional file 1. Supplementary Material 1: Check list PRISMA. Supplementary Material 2: Research Algorithm for RCT & Protocols. Supplementary Material 3: Characteristics of included studies. Supplementary Material 4: Distribution of terms used. Supplementary Material 5: Risk of bias assessment. [file 13054_2023_4637_MOESM1_ESM.docx]
